# Supplementary material for: Titanium-based potassium-ion battery positive electrode with extraordinarily high redox potential
Source: Nat Commun. 2020 Mar 20;11:1484. doi: 10.1038/s41467-020-15244-6 (PMC7083823; doi:10.1038/s41467-020-15244-6)
Supplement: Supplementary file 1 — Supplementary Information [file 41467_2020_15244_MOESM1_ESM.pdf]

Supplementary information

Titanium-based potassium-ion battery positive electrode with extraordinarily high redox potential

Fedotov et al.

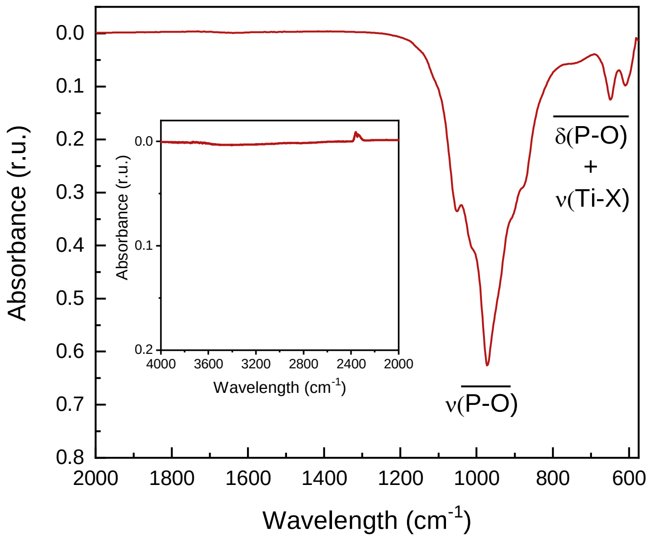

Supplementary Fig. 1. Mid-IR spectrum of $KTiPO_4F$ within the 4000–600 cm^{-1} range. Inset: enlarged 4000–2000 cm^{-1} region.

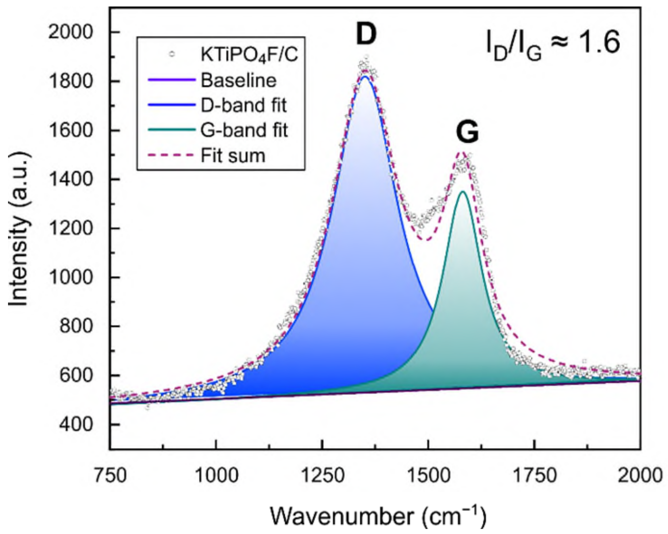

Supplementary Fig. 2. Raman spectrum of the $KTiPO_4F/C$ composite in the range of 750–2000 cm^{-1}. The D and G bands each fitted with a single Lorentzian ($I_D/I_G \approx 1.6$).

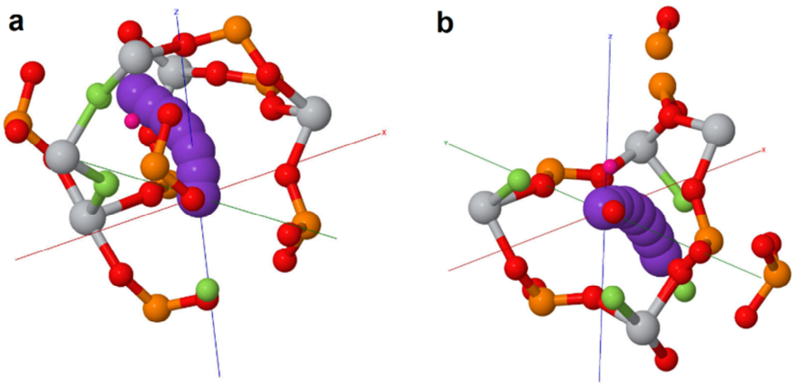

Supplementary Fig. 3. Two considered non-equivalent K–K transitions of the K migration pathway along the *c* axis.

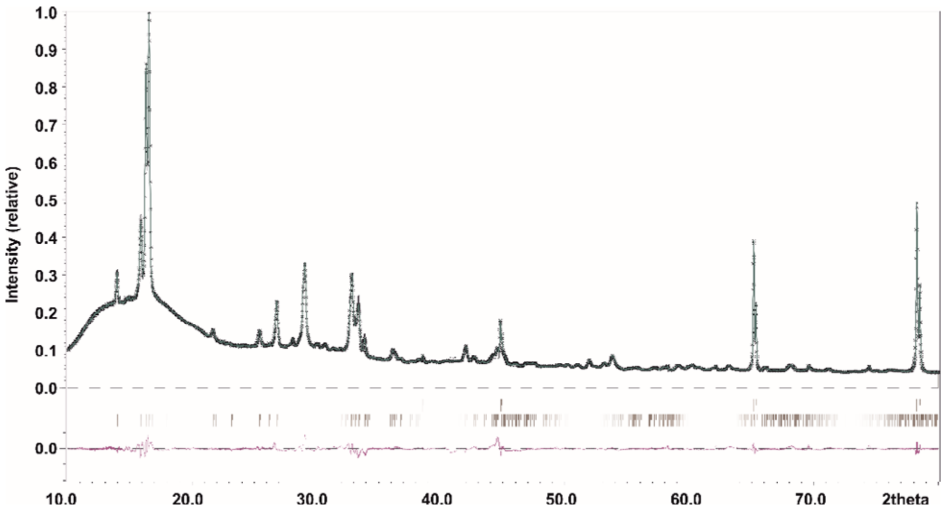

Supplementary Fig. 4. Experimental, calculated and difference XRD patterns after Rietveld refinement of $K_{0.19(2)}TiPO_4F$. Bragg reflections for $K_{0.19(2)}TiPO_4F$ and Al support are denoted as brown and dark-grey bars respectively. Intensities are given in relative units.

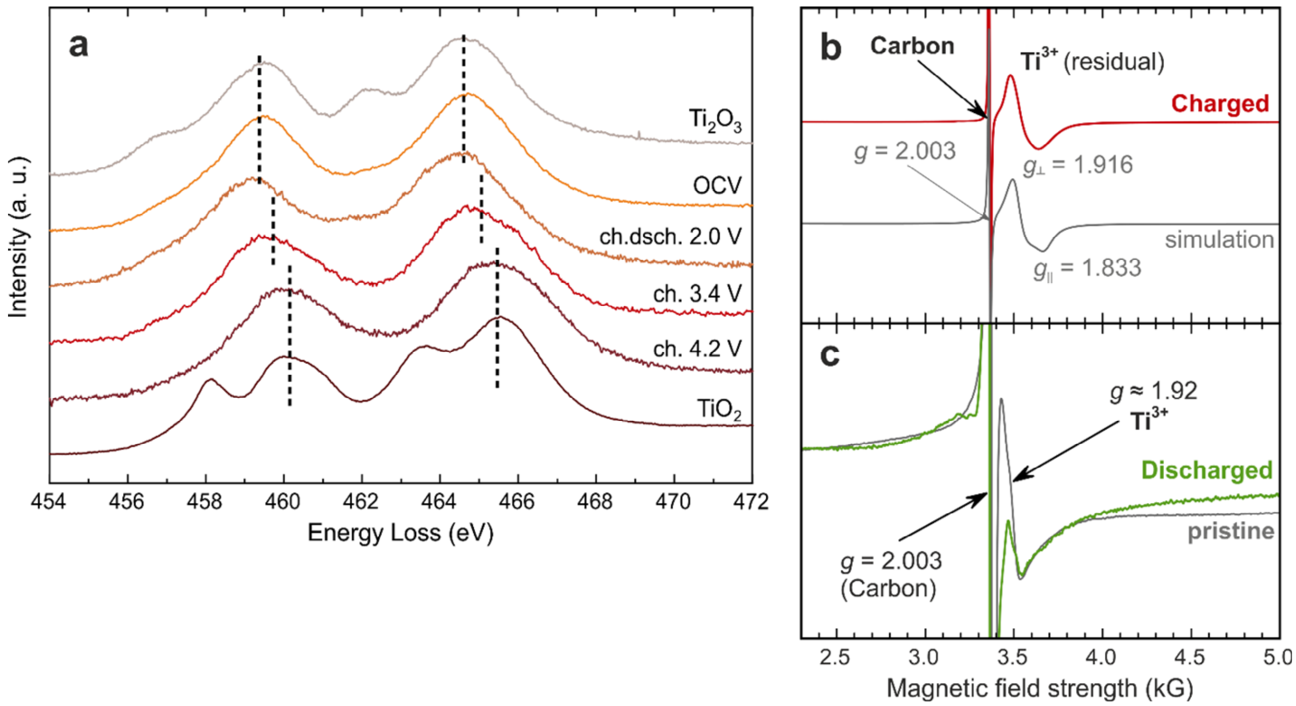

Supplementary Fig. 5. Titanium valence state characterization. **a** EELS spectra of recovered electrode materials. Ti_2O_3 and TiO_2 EELS spectra are given for reference. Note that the fine structure of the spectra might be not distinct enough due to specifics of EELS spectra acquisition on cycled electrodes. **b** Experimental (*ex-situ*) and simulated EPR spectra for the $KTiPO_4F$/C electrode charged to 4.2 V. **c** Experimental (*ex-situ*) EPR spectra of initial and discharged to 2.0 V (after charge to 4.2 V) $KTiPO_4F$/C electrodes.

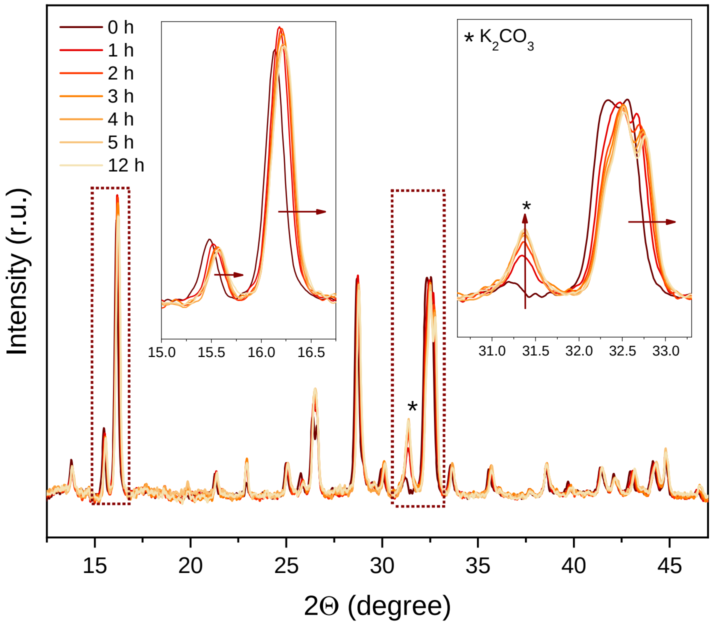

Supplementary Fig. 6. Time-resolved XRD patterns of the $KTiPO_4F$/C electrode material exposed to air. Insets: the shift of the peaks confirms unit cell shrinkage due to Ti oxidation and K^+ loss. Asterisk (*) designates the peak of K_2CO_3.

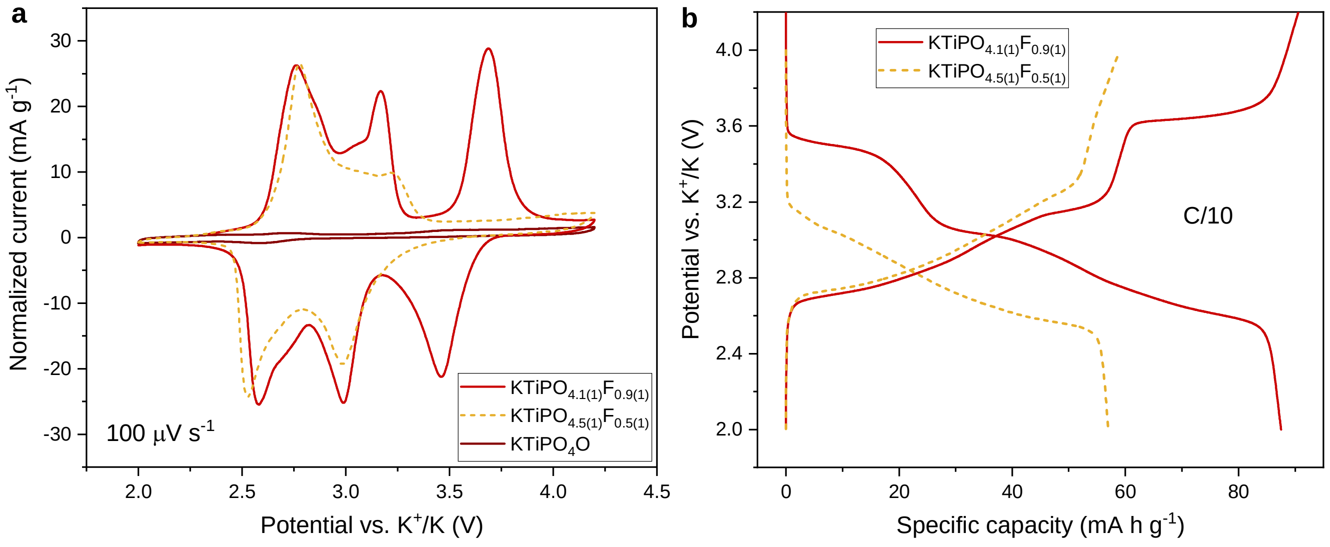

Supplementary Fig. 7. Electrochemical measurements of $KTiPO_{4+\delta}F_{1-\delta}$. **a** CV curves for $KTiPO_4F$ ($KTiPO_{4.1(1)}F_{0.5(1)}$), $KTiPO_{4.5(1)}F_{0.5(1)}$ and $KTiPO_4O$. **b** Galvanostatic curves for $KTiPO_{4.1(1)}F_{0.5(1)}$ and $KTiPO_{4.5(1)}F_{0.5(1)}$. $KTiPO_{4.5(1)}F_{0.5(1)}$ was obtained by adjusting the $Ti_{(metallic)}/TiOSO_4$ ratio during synthesis. The fluorine content was determined by SEM-EDX (in the same manner as for $KTiPO_4F$).

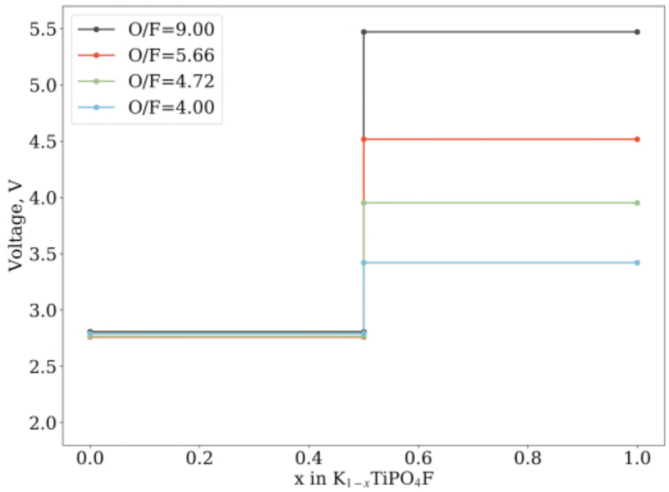

Supplementary Fig. 8. DFT+U calculated average intercalation potentials. $K_1TiPO_{4+\delta}F_{1-\delta}/K_{0.5}TiPO_{4+\delta}F_{1-\delta}$ and $K_{0.5}TiPO_{4+\delta}F_{1-\delta}/TiPO_{4+\delta}F_{1-\delta}$ transitions at different F:O ratios (δ = 0, 0.125, 0.25, and 0.5).

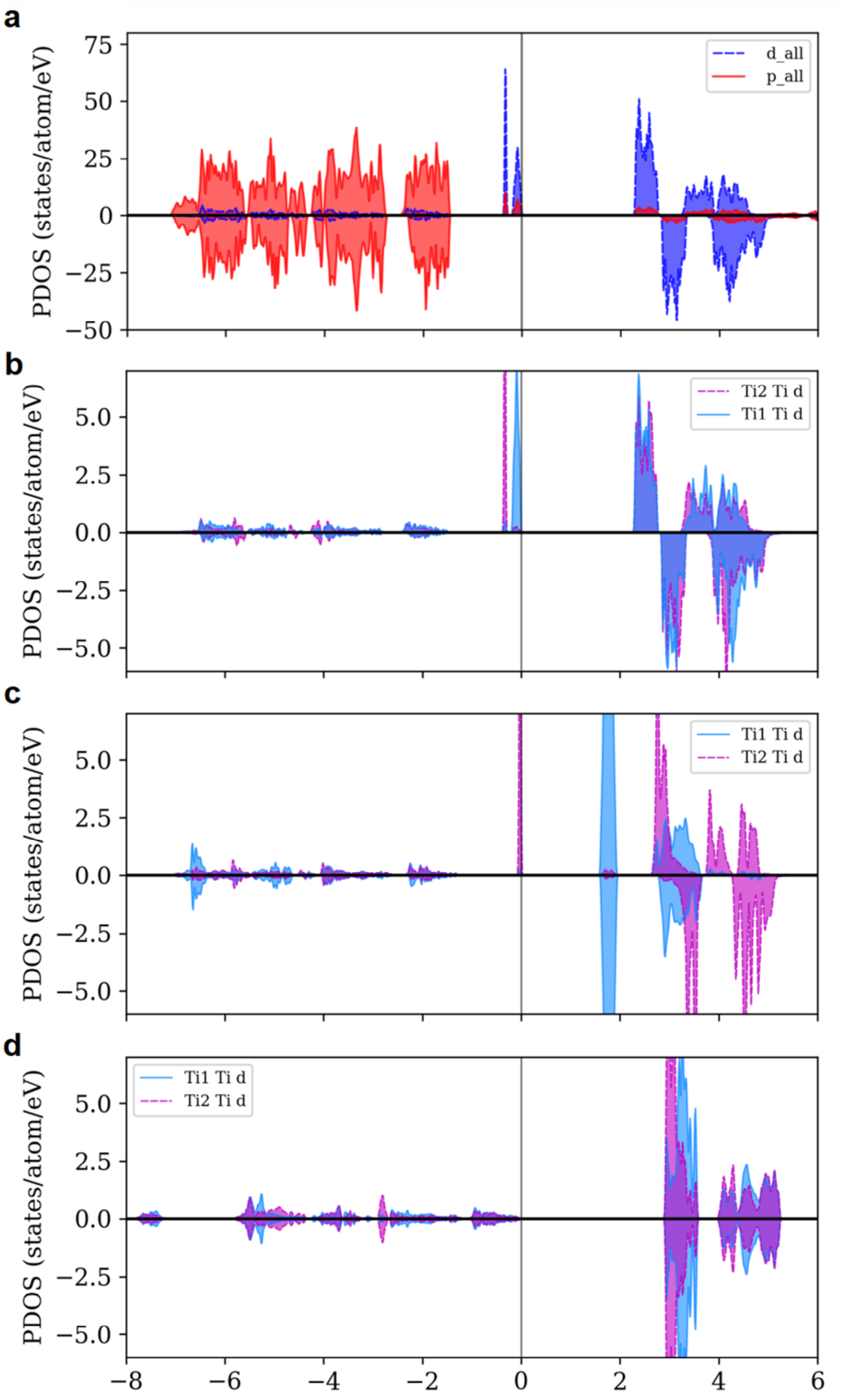

Supplementary Fig. 9. Local partial density of states (pDOS) calculated with DFT+U. Sum over all atoms for *p* (oxygen) and *d* (titanium) orbitals for $KTiPO_4F$ (**a**). Local pDOS for *d*-orbitals at Ti1 (*cis*) and Ti2 (*trans*) atoms for $KTiPO_4F$ (**b**), $K_{0.5}TiPO_4F$ (**c**), and $TiPO_4F$ (**d**), respectively. The vertical line designates the Fermi level. Gaussian smearing is applied.

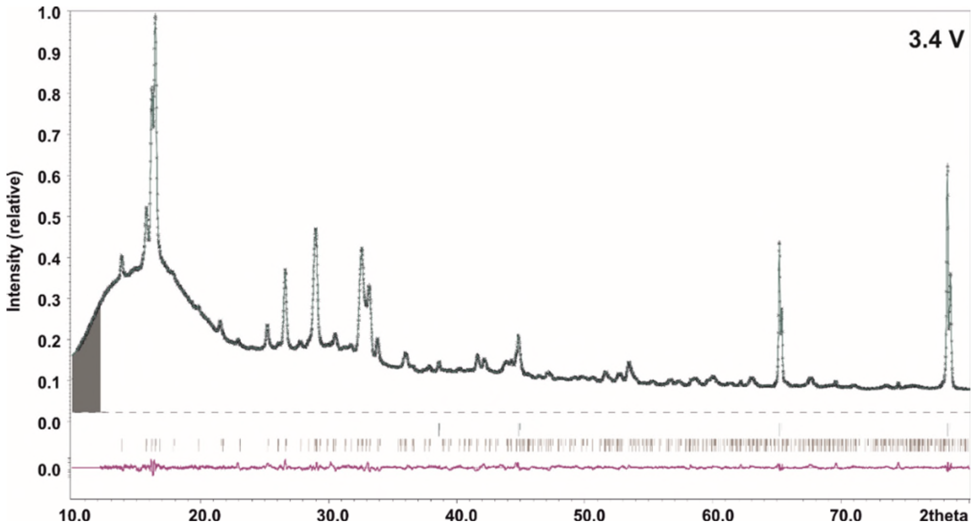

Supplementary Fig. 10. Experimental, calculated and difference XRD patterns after Rietveld refinement of $K_{0.46(2)}TiPO_4F$. Bragg reflections for $K_{0.46(2)}TiPO_4F$ and Al support are denoted as brown and dark-grey bars respectively. Intensities are given in relative units.

Supplementary Table 1. Crystallographic data and parameters of the Rietveld refinement for $KTiPO_4F$.

Formula	$KTiPO_4F$
Space group	$Pna2_1$
a, Å	13.0020(2)
b, Å	6.43420(8)
c, Å	10.7636(2)
V, Å^3	900.45(2)
Z	8
Radiation	X-rays, Cu-K_α, λ_1 = 1.54051 Å, λ_2 = 1.54433 Å
2θ range, step, deg.	10–120, 0.015
GOF	1.62
R_F, %	2.44
R_p, R_{wp}, %	3.99, 5.22

Supplementary Table 2. Atomic positions, fractional coordinates and isotropic displacement parameters for $KTiPO_4F$.

Atom	Position	*x/a*	*y/b*	*z/c*	$U_{iso,}$ Å^2	Occupancy
K1	4d	0.3789(5)	0.7798(9)	0.3045(8)	0.016(2)	0.732(7)
K1'	4d	0.3999(17)	0.830(3)	0.4330(19)	0.016(2)	0.268(7)
K2	4d	0.086(2)	0.632(5)	–0.012(3)	0.033(3)	0.151(10)
K2'	4d	0.0875(13)	0.703(3)	0.158(2)	0.033(3)	0.264(13)
K2"	4d	0.1051(8)	0.7258(17)	0.0678(12)	0.033(3)	0.585(17)
Ti1	4d	0.3867(2)	0.4951(9)	0.0022(7)	0.0255(6)	1
Ti2	4d	0.2448(4)	0.2574(10)	0.2564(9)	0.0255(6)	1
P1	4d	0.5002(7)	0.3292(5)	0.2573(11)	0.0157(8)	1
P2	4d	0.1798(3)	0.5039(15)	0.5054(13)	0.0157(8)	1
O1	4d	0.4758(7)	0.4903(18)	0.1545(11)	0.0080(12)	1
O2	4d	0.4981(12)	0.4567(17)	0.3803(11)	0.0080(12)	1
O3	4d	0.4045(8)	0.2017(18)	0.2804(16)	0.0080(12)	1
O4	4d	0.5953(8)	0.1893(18)	0.2386(14)	0.0080(12)	1
O5	4d	0.1220(10)	0.3082(16)	0.5454(13)	0.0080(12)	1
O6	4d	0.1066(10)	0.6862(16)	0.4842(14)	0.0080(12)	1
O7	4d	0.2574(10)	0.540(3)	0.6112(13)	0.0080(12)	1
O8	4d	0.2459(11)	0.463(3)	0.3889(12)	0.0080(12)	1
F1	4d	0.2706(12)	0.534(3)	0.8845(13)	0.0208(19)	1
F2	4d	0.2752(11)	0.494(3)	0.1308(12)	0.0208(19)	1

Supplementary Table 3. Selected interatomic distances for $KTiPO_4F$.

Bond	Distance, Å	Bond	Distance, Å
Ti1–O1	2.007(13)	Ti2–O3	2.123(11)
Ti1–O2	2.015(15)	Ti2–O4	1.982(12)
Ti1–O5	2.071(12)	Ti2–O7	2.098(18)
Ti1–O6	1.999(12)	Ti2–O8	1.946(18)
Ti1–F1	1.986(16)	Ti2–F1	2.005(18)
Ti1–F2	2.004(15)	Ti2–F2	2.075(18)
P1–O1	1.550(14)	P2–O5	1.528(14)
P1–O2	1.558(15)	P2–O6	1.527(14)
P1–O3	1.511(13)	P2–O7	1.539(17)
P1–O4	1.543(13)	P2–O8	1.543(18)

Supplementary Table 4. Space group and lattice parameters of different phases calculated with DFT.

Structure	*a*, Å	*b*, Å	*c*, Å	*V*, Å^3	S.G.
$KTiPO_4F$	6.56	11.04	13.30	963.4	$Pna2_1$
$K_{0.5}TiPO_4F$	6.41	10.90	13.29	928.6	$Pna2_1$
$TiPO_4F$	6.32	10.83	12.91	883.5	*Pnan*

Supplementary Table 5. SEM-EDX data and processing of O:F ratio in $KCrPO_4F$ (reference) and $KTiPO_4F$.

	$KCrPO_4F$				$KTiPO_4F$			
Point	O	F	O/(O+F)*	F/(O+F)	O	F	O/(O+F)	F/(O+F)
1	48.52	12.02	4.01	0.99	39.23	9.69	4.01	0.99
2	36.65	9.08	4.01	0.99	28.19	5.83	4.14	0.86
3	45.75	12.14	3.95	1.05	44.09	10.33	4.05	0.95
4	46.87	13.4	3.89	1.11	27.48	5.52	4.16	0.84
5	50.33	14.95	3.85	1.15	38.70	9.00	4.06	0.94
6	47.13	12.56	3.95	1.05	31.99	6.64	4.14	0.86
7	44.13	10.53	4.04	0.96	43.69	11.40	3.97	1.03
8	40.9	10.27	4.00	1.00	30.86	7.38	4.04	0.96
9	42.04	9.92	4.05	0.95	47.18	12.72	3.94	1.06
10	36.31	8.19	4.08	0.92	47.39	11.10	4.05	0.95
11	37.37	8.47	4.08	0.92	47.31	12.75	3.94	1.06
12	50.59	14.86	3.86	1.14	29.53	6.03	4.15	0.85
13	46.96	12.83	3.93	1.07	46.57	10.76	4.06	0.94
14	48.19	14.15	3.87	1.13	34.63	7.86	4.08	0.92
15	49.94	13.12	3.96	1.04	40.44	8.30	4.15	0.85
**Average**			**3.97**	**1.03**			**4.06**	**0.94**
**Std**			0.08	0.08			0.08	0.08
**Normalized average**							**4.10**	**0.91**

* – ratio normalized per formula unit given that O + F = 5

Supplementary Table 6. Crystallographic data and parameters of the Rietveld refinement for $K_{0.19(2)}TiPO_4F$.

Formula	$K_{0.19(2)}TiPO_4F$ (4.2 V)
Space group	*Pnan*
a, Å	12.7460(5)
b, Å	6.2454(2)
c, Å	10.6124(6)
V, Å^3	844.80(7)
Z	8
Radiation	X-rays, Cu-K_α, λ_1 = 1.54051 Å, λ_2 = 1.54433 Å
2θ range, step, deg.	10–80, 0.015
GOF	0.14
R_p, R_{wp}, %	2.62, 3.93

Supplementary Table 7. Atomic positions, fractional coordinates and occupancies for $K_{0.19(2)}TiPO_4F$.

Atom	Position	*x/a*	*y/b*	*z/c*	Occupancy
K1	8d	0.861(5)	0.107(8)	0.657(4)	0.19(2)
Ti1	4d	0	0	0	1
Ti2	4d	0.1481(6)	0.25	0.75	1
P1	4d	0.25	0.0673(16)	0	1
P2	4d	–0.0675(8)	0.25	0.25	1
O1	8d	0.240(2)	0.227(3)	–0.110(2)	1
O2	8d	0.1452(10)	–0.042(3)	0.036(2)	1
O3	8d	–0.129(2)	0.039(3)	0.279(3)	1
O4	8d	0.0007(19)	0.268(8)	0.371(2)	1
F1	8d	0.015(2)	0.288(7)	0.612(3)	1

Supplementary Table 8. Crystallographic data and parameters of the Rietveld refinement for $K_{0.46(2)}TiPO_4F$.

Formula	$K_{0.46(2)}TiPO_4F$ (3.4 V)
Space group	$Pna2_1$
a, Å	12.8853(4)
b, Å	6.29853(18)
c, Å	10.6407(5)
V, Å^3	863.58(5)
Z	8
Radiation	X-rays, CuK_α,
	λ_1 = 1.54051 Å, λ_2 = 1.54433 Å
2θ range, step, deg.	10–80, 0.015
GOF	0.07
R_F, %	4.15
R_p, R_{wp}, %	1.45, 2.05

Supplementary Table 9. Atomic positions, fractional coordinates and isotropic displacement parameters for $K_{0.46(2)}TiPO_4F$.

Atom	Position	*x/a*	*y/b*	*z/c*	Occupancy
K1	4d	0.4055(7)	0.8440(10)	0.4254(11)	0.718(7)
K2	4d	0.586(2)	–0.210(4)	0.174(3)	0.204(8)
Ti1	4d	0.3897(4)	0.5004(19)	–0.0103(8)	1
Ti2	4d	0.2533(6)	0.2442(15)	0.2374(9)	1
P1	4d	0.5136(5)	0.3289(7)	0.2362(9)	1
P2	4d	0.1875(4)	0.503(2)	0.4748(8)	1
O1	4d	0.4993(8)	0.475(2)	0.1216(11)	1
O2	4d	0.5356(8)	0.464(2)	0.3538(11)	1
O3	4d	0.4092(8)	0.223(2)	0.2689(15)	1
O4	4d	0.6055(10)	0.178(2)	0.2127(16)	1
O5	4d	0.1129(11)	0.316(2)	0.4987(13)	1
O6	4d	0.1243(12)	0.706(2)	0.4491(15)	1
O7	4d	0.2565(10)	0.537(4)	0.5913(12)	1
O8	4d	0.2455(12)	0.467(3)	0.3501(12)	1
F1	4d	0.2815(11)	0.525(5)	–0.1443(12)	1
F2	4d	0.2639(13)	0.462(4)	0.1048(16)	1

Supplementary notes

Air/moisture sensitivity

Since Ti^{3+} generally displays poor stability when exposed to moisture or air, dealing with $KTiPO_4F$ requires inert (or reducing) conditions at each step to preserve its chemical composition and Ti^{3+} oxidation state. A Ti^{3+}-to-Ti^{4+} conversion in $KTiPO_4F$ can take place according to the following possible reactions:

$KTi^{3+}PO_4F + x/2H_2O + x/4O_2$ (from air) → $KTi^{(3+x)+}PO_4F_{1-x}O_x + xHF$

$KTi^{3+}PO_4F + y/2H_2O + y/4O_2$ (from air) → $K_{1-y}Ti^{(3+y)+}PO_4F + yKOH$ with further catching of CO_2 from air by KOH to form K_2CO_3.

Both presumable reactions lead to formation of Ti^{4+}-enriched materials showing reduced cell parameters. In the second case formation of K_2CO_3 can be easily observed with a time-dependent XRD of samples exposed to air (Supplementary Fig. 6).

In summary, the overall degradation reaction can be written as follows:

$KTi^{3+}PO_4F + (x+y)/2O_2 + y/2CO_2$ (from air) → $K_{1-y}Ti^{(3+x+y)+}PO_4F_{1-x}O_x + xKF + y/2K_2CO_3$

As seen, water may not even take part in the degradation mechanism of $KTi^{3+}PO_4F$ as evidenced by the $KTiPO_4F$ stability under the synthesis conditions (highly acidic medium, pH ~1–2, reducing conditions created by hydrogen from dissolving of metallic Ti) as well as in pure degassed (deoxygenated) water.
